# A multifaceted microenvironment nanoregulator for targeted ovarian cancer therapy

**DOI:** 10.3389/fphar.2025.1584463

**Published:** 2025-03-27

**Authors:** Yizheng Zu, Min Li, Ruyue Li, Shaohan Ma, Yu’e Yang, Shun Zhang, Yuan Ma, Tiantian Wu, Chunfang Ha

**Affiliations:** ^1^ General Hospital of Ningxia Medical University, Yinchuan, China; ^2^ Key Laboratory of Fertility Preservation and Maintenance of Ministry of Education, Ningxia Medical University, Yinchuan, China; ^3^ School of Pharmacy, Hainan Medical University, Haikou, China

**Keywords:** plant-derived exosomes, tumor microenvironment, curcumin, targeted therapy, drug delivery

## Abstract

The treatment of ovarian cancer is hindered by its insidious onset and rapid progression. Exosomes (EXOs) present a promising therapeutic strategy for ovarian cancer by modulating the tumor microenvironment. However, concerns regarding the biosafety of animal-derived EXOs pose significant challenges to the development of innovative formulations. In this study, we propose a universal strategy to engineer plant-derived EXOs as microenvironment nanoregulators for targeted ovarian cancer therapy. EXOs derived from ginger were purified, loaded with the natural bioactive compound curcumin (Cur) with high encapsulation efficiency, and functionalized with a tumor-targeting aptamer. Upon intravenous administration, the resulting multifaceted microenvironment nanoregulator, termed ^A^GE@Cur, effectively accumulates at the tumor site and exerts a tumor-suppressive effect through remodeling the tumor microenvironment. This novel therapeutic platform not only addresses the limitations of animal-derived EXOs but also paves the way for the development of innovative microenvironment regulators in clinical applications.

## 1 Introduction

Ovarian cancer, a leading cause of mortality among gynecological malignancies, represents a significant public health challenge, driven by its high mortality rate and the absence of effective screening methods for early detection (Matulonis et al., 2016; Webb and Jordan, 2024). It constitutes a substantial proportion of gynecological cancers and remains a major cause of cancer-related deaths among women globally. The disease’s heterogeneity and complexity, combined with its insidious onset and rapid progression, present formidable obstacles to effective treatment strategies. Despite advancements in surgical techniques and conventional chemotherapy, the overall 5-year survival rate for patients with advanced-stage ovarian cancer has shown limited improvement (Webb and Jordan, 2024). This underscores the urgent need for novel therapeutic approaches capable of enhancing treatment efficacy, improving patient outcomes, and alleviating the severe side effects associated with current therapies. The exploration of new therapeutic agents, particularly through targeted therapies and combination treatment regimens, is critical in the fight against ovarian cancer (Webb and Jordan, 2024; Vázquez-García et al., 2022; Wang et al., 2024). Immunotherapy and the tumor microenvironment may emerge as pivotal targets, offering the potential to develop more effective and less toxic treatment options for those affected by this devastating disease. Nanocarriers represent a cutting-edge technology in the field of drug delivery, offering a sophisticated approach to enhance the efficacy and safety of therapeutic agents. These nano-sized vehicles are engineered to encapsulate, protect, and transport a diverse array of pharmaceuticals, including small molecules, peptides, proteins, and nucleic acids, to their target sites within the body (Wang et al., 2024; Zhao P. et al., 2024).

Exosomes (EXOs) are nanoscale extracellular vesicles secreted by various cell types and have attracted significant attention within the oncology research community due to their potential roles in tumor progression, metastasis, and intercellular communication (Théry et al., 2002; Liu and Su, 2019; Kalluri and LeBleu, 2020). The therapeutic potential of EXOs is increasingly recognized, as they offer a unique platform for targeted drug delivery and immunomodulation in cancer treatment. EXOs can carry a wide array of biological molecules, including proteins, lipids, and microRNAs, all of which can influence key processes such as tumor growth inhibition, angiogenesis, and immune evasion (Andre et al., 2024; Cheng and Kalluri, 2023). EXOs derived from normal cells or engineered with therapeutic agents have shown promise in inhibiting tumor growth and altering the tumor microenvironment. However, exosomes derived from animal sources, particularly stem cell-derived extracellular vesicles, present several inherent limitations that hinder their broader application in disease therapy and clinical translation. Notably, animal-derived EXOs pose a significant risk of immunogenicity. The presence of xenogeneic antigens on their surface can provoke an immune response in humans, leading to inflammation, rejection, and potential adverse effects. Additionally, the biological activity and functionality of animal-derived EXOs may not be fully retained when transferred to human systems. Furthermore, the high costs associated with the isolation and preparation of EXOs remain a significant barrier to their widespread research and clinical use (Cheng and Kalluri, 2023; Robbins and Morelli, 2014). In contrast, plant-derived exosomes (PDEs) offer a promising solution to these challenges. The isolation and characterization of PDEs have gained considerable attention in biotechnology, as plants can be cultivated in large quantities, offering a cost-effective and scalable means of production, free from the ethical concerns tied to animal-based sources (Liang et al., 2025; Teng et al., 2025). The unique properties of PDEs position them as a versatile and innovative platform for the development of novel therapeutics and diagnostic tools.

**FIGURE 1:**
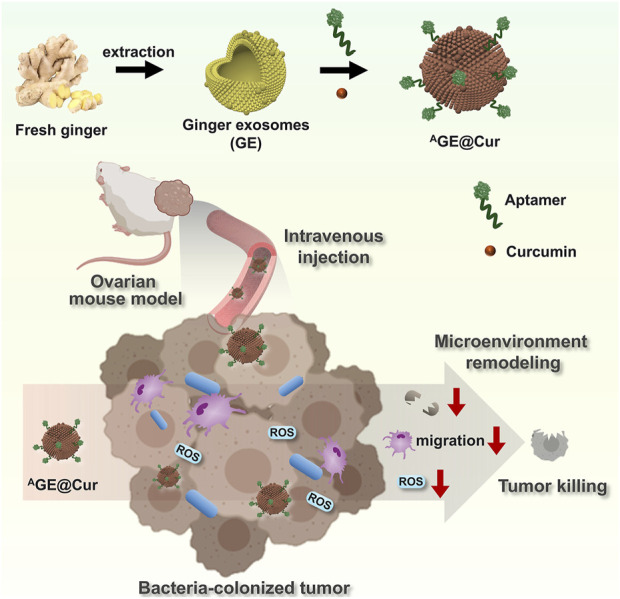
Schematic illustration of the construction of Plant-derived exosomes-based microenvironment nanoregulator ^A^GE@Cur and its application in ovarian cancer therapy. ROS: reactive oxygen species. The pink cell represents macrophage. The blue cell represents bacteria.

In this study, we have developed a tumor-targeted microenvironment nanoregulator based on plant-derived exosomes (PDEs) extracted from ginger, a commonly consumed vegetable and a traditional Chinese medicinal herb (Figure 1). Curcumin (Zhong et al., 2022; Fu et al., 2021), a potent anticancer agent, is encapsulated within the PDEs to facilitate tumor inhibition, intratumoral bacterial eradication, and reactive oxygen species (ROS) scavenging. To enable targeted therapy for ovarian cancer, we modified the surface of the ginger-derived PDEs (GE) with a tumor-targeting aptamer, thereby constructing the therapeutic platform ^A^GE@Cur. Through comprehensive and systematic characterization of ^A^GE@Cur, we confirmed that this rationally designed nanoplatform exhibits high encapsulation efficiency, outstanding biosafety, and remarkable targetability, demonstrating effective antitumor activity in a mouse model. ^A^GE@Cur significantly enhances ROS clearance, suppresses intratumoral bacterial growth, and exerts immune regulatory effects, collectively modulating the tumor microenvironment and inducing substantial tumor apoptosis. Our study demonstrates the first example of PDEs-based multifunctional nanoplatform with aptamer modification, which is developed for targeted tumor microenvironment intervention-based cancer therapy.

## 2 Materials and methods

### 2.1 Materials

Curcumin was purchased from MedChemExpress (MCE, China). Chemicals and solvents were obtained commercially and used without further purification. Millipore water was used to prepare aqueous solutions. Annexin V-FITC/PI Apoptosis Detection Kit (cat# 40302ES60), purchased from Yeasen, Shanghai, China. Hoechst 33342 and cell count kit-8 (CCK-8) were purchased from Solarbio kit (Beijing, China). Serum-Free Cell Freezing Medium (6032011) were purchased from Shenzhen Dakewe Bioengineering co., Ltd. ID8 cell was purchased from the American Type Culture Collection. The ID8 cells were maintained in DMEM supplemented with 10% FBS and penicillin/streptomycin in a humidified atmosphere containing 5% CO_2_ at 37°C.

### 2.2 Isolation and purification of the ginger-derived PDEs

The isolation and purification of the ginger-derived PDEs were followed the reported protocol with some modification (Xie et al., 2024; Guo et al., 2025; Cui et al., 2024). Briefly, fresh ginger rhizomes were washed with Millipore water, dried with tissues, and cut into small pieces. The prepared ginger pieces were homogenized in a cold PBS buffer. The mixture was subjected to a series of centrifugations at 4°C to remove debris and large cellular components. The filtered extract was then concentrated using an ultrafiltration device with a 100 kDa molecular weight. The extracted GE were characterized using electron microscopy for DLS, transmission electron microscope (TEM), and atomic force microscope for size distribution.

### 2.3 Drug loading and entrapment efficiency

The Cur was loaded into GE by magnetic stirring. Then, it was subjected to ultracentrifugation for 0.5 h at 100,00 g. The amount of drug load and drug loading efficiency (DLE) within GE were quantified using a spectrophotometer (UV-2600, SHIMADZU, Kyoto, Japan). The absorbance of Cur is 420 nm. The DLE was calculated according to the following Equations:

$$\%\text{ DLE}=\left[\left(\text{Drug added}-\text{unloaded Drug}\right)/\text{ Drug added}\right]\times 100$$

### 2.4 Evaluation of Cur release

The ^A^GE@Cur was dispersed into PBS with varying pH (6.0, 7.4, and 8.3). Subsequently, the fraction was sealed within a dialysis cassette with a molecular weight cutoff of 5 kDa. The dialysis cassette was then submerged in 10 mL of PBS, matched to the respective pH, and incubated under continuous agitation at 37°C. At predetermined time points, a 1 mL aliquot of the dialysate was withdrawn from each sample, and an equal volume of the corresponding PBS buffer was replenished to the remaining dialysate to maintain the volume. The extracted dialysate samples were subjected to analysis utilizing a UV spectrophotometer.

### 2.5 Cellular cytotoxicity assay

To assess cellular viability, the Cell Counting Kit-8 (CCK-8) assay was employed according to the following experimental protocol. ID8 cells were seeded into a 96-well plate at a density of 5,000 cells per well and allowed to adhere overnight in an incubator set to 37°C with 5% CO_2_. After adhesion the culture medium was replaced with fresh medium containing varying concentrations of the test drugs. After incubation for 48 h, the medium was aspirated carefully, and 100 µL of fresh culture medium containing 10% CCK-8 solution was added to each well. Subsequently, the absorbance of each well was measured at 450 nm using a microplate reader, with a reference wavelength of 630 nm to correct for optical density.

### 2.6 Animals and tumor model-building

Female BALB/c mice, aged 6 weeks, were procured from Beijing Vital River Laboratory Animal Technology Co., Ltd. All experimental procedures involving animals were conducted in accordance with the guidelines approved by the Ethics Review Committee of Ningxia Medical University General Hospital (NO. KYLL-2022-1241). To build the mouse tumor model with intratumoral bacteria, an intratumoral injection of *Escherichia coli* (1 × 10^6^ CFU in 10 μL) was performed when the tumor volume reached 100 mm^3^.

### 2.7 Statistical analysis

Data represent the mean ± s.d. from indicated independent replicates. Statistical analysis was conducted using GraphPad Prism. For comparisons between two groups, means were compared using the unpaired two-tailed Student’s t-test. A value of P < 0.05 was considered statistically significant.

## 3 Results and discussion

### 3.1 Preparation and characterization of ^A^GE@Cur

**FIGURE 2:**
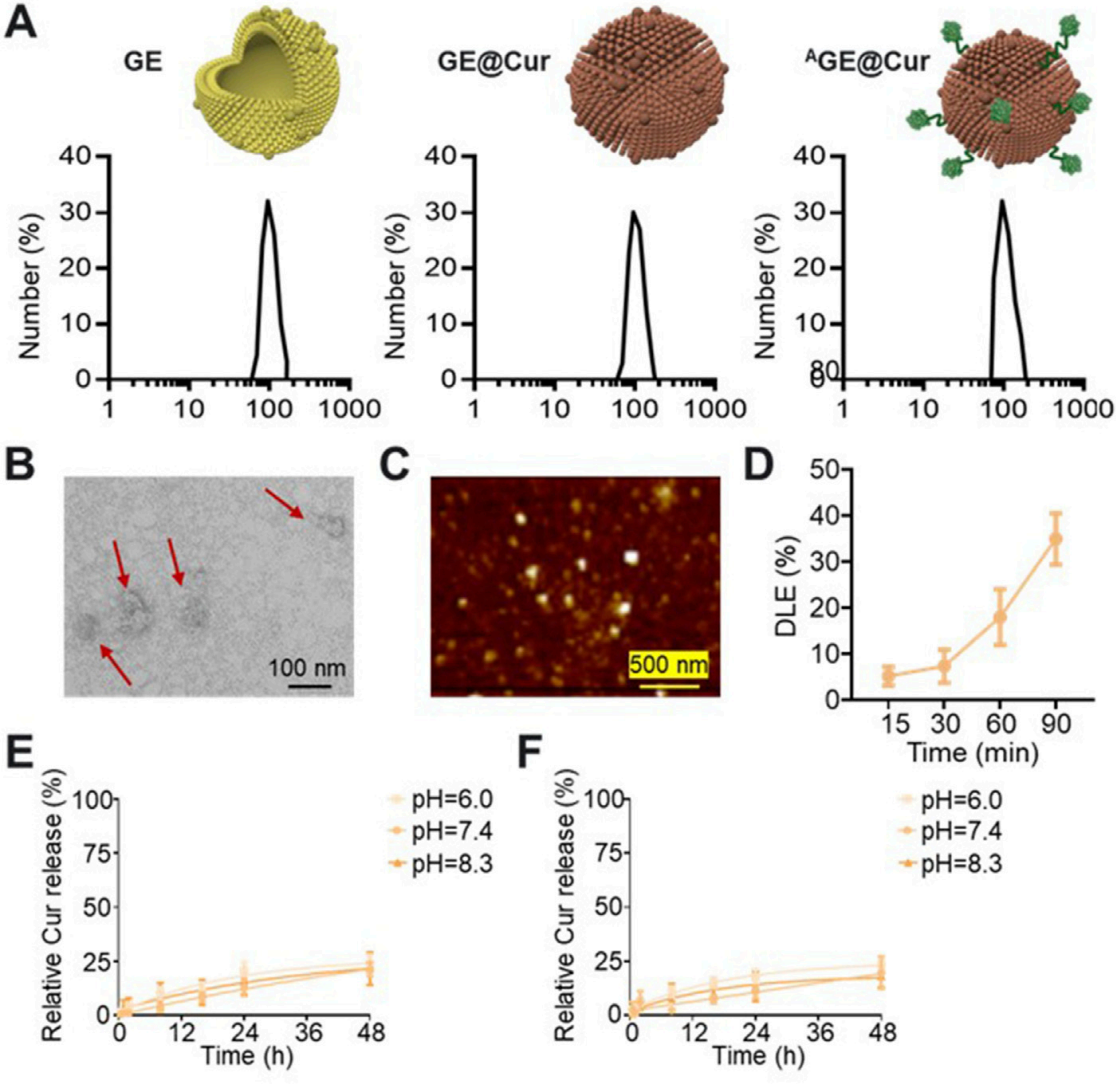
**(A)** Hydrodynamic sizes analysis of the GE, GE@Cur, and ^A^GE@Cur. **(B)** TEM image of the ^A^GE@Cur. Scale bar: 100 nm. **(C)** AFM image of the ^A^GE@Cur. Scale bar: 500 nm. **(D)** Drug loading efficacy (DLE) of Cur loaded in the ^A^GE@Cur after different time of incubation. **(E)** Time dependent Cur release curve of GE@Cur in PBS with different pH. **(F)** Time dependent Cur release curve of ^A^GE@Cur in PBS with different pH. Data are expressed as mean ± SEM of three independent experiments.

We constructed the PDEs-based microenvironment nanoregulator using ginger-derived GE as active agents and drug carrier. As reported, nano-sized extracellular vesicles are known to encapsulate a plethora of bioactive compounds, including gingerols, shogaols, and other phenolic substances, that are inherent to the ginger rhizome (Cui et al., 2024; Bahri et al., 2024). GE exhibits a range of therapeutic properties, such as anti-inflammatory, antioxidant, and anticancer effects, which have been attributed to the molecular constituents within these vesicles (Bahri et al., 2024; Zhao B. et al., 2024). Ginger-derived exosome-like nanoparticles as feasible therapeutic nano-agents against diseases. The purified GE in our study was characterized with uniform morphology and the diameter of the GE is about 120.7 ± 5.8 nm (Figure 2A; Supplementary Figure S1). After the drug loading of Cur, the diameter of the nanoparticles was slightly increase (GE@Cur: 125.7 ± 7.1 nm). To achieve targeted therapy, the tumor targeted aptamer AS1411 conjugated with cholesterol was utilized for GE modification. The AS1411 spontaneously binding to the surface of the GE. The targeted microenvironment nanoregulator ^A^GE@Cur, with the diameter of 140.9 ± 11.1 nm, exhibited uniform and regular morphology without any aggregation based on the morphological assessment as shown in Figures 2B, C. In drug loading, UV absorption analysis at 420 nm revealed that the 1.5 h incubation reached ideal DLE of 36.8% (Figure 2D). Subsequently, the drug release efficacy of Cur was investigated to evaluate the delivery performance in PBS with different pH. As shown in Figures 2E, F, prolonged release profile, characterized by a time-dependent release kinetics, achieving a cumulative release of 25% over a 48-h period without sudden release. Overall, the ^A^GE@Cur would be favorable for ovarian cancer treatment.

### 3.2 *In vitro* antitumor activity evaluation

**FIGURE 3:**
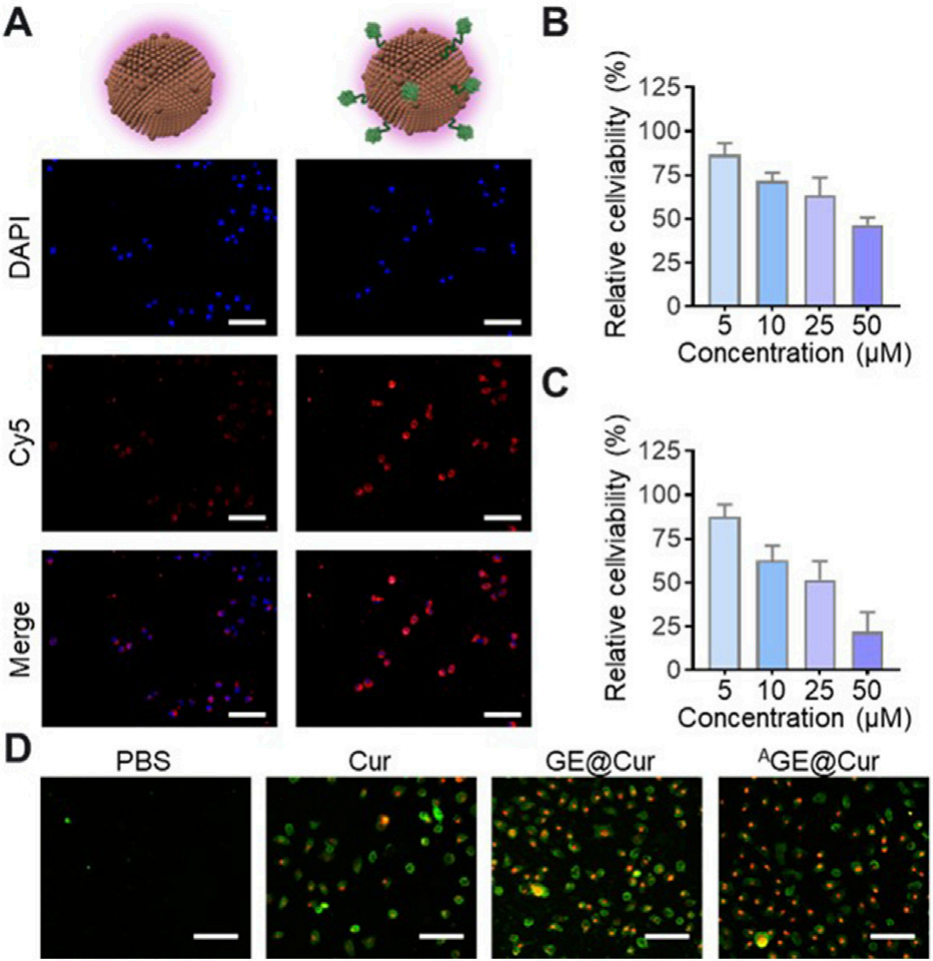
**(A)** Confocal images of ID8 cells cultured with Cy5-labeled GE@Cur or ^A^GE@Cur for 0.5 h at 37 °C. Scale bar: 100 μm. **(B)** Relative cell viability of ID8 cell after the treatment of different concentration of GE@Cur. **(C)** Relative cell viability of ID8 cell after the treatment of different concentration of ^A^GE@Cur. **(D)** Cell apoptosis analysis of cells after the treatment of PBS, Cur, GE@Cur, and ^A^GE@Cur. Scale bar: 100 μm. The drug concentration was based on 5 µM of Cur. Data are expressed as mean ± SEM of three independent experiments.

To verify the targetability of the ^A^GE@Cur, fluorescence probe Cyanine 5 (Cy5) was introduced for confocal images analysis. As shown in Figure 3A, significantly enhanced fluorescence signal was detected in the ^A^GE@Cur treatment group. To evaluate the potential inhibition effect, we first investigated the cytotoxicity of Cur, GE@Cur, and ^A^GE@Cur on ID8 cells by CCK8 assay (The concentration of the free Cur was the same as that of Cur loaded in PDEs). ID8 cells treated with Cur, GE@Cur, and ^A^GE@Cur showed dose dependent cytotoxicity (Figures 3B, C; Supplementary Figure S2). As expected, remarkable enhanced effect in cell inhibition was achieved by ^A^GE@Cur compared with GE@Cur, which indicates the modification of the targeted ligand is beneficial for the formulations to exert therapeutic effects in cancer therapy (Supplementary Figure S3). In addition, the cytotoxicity of the drug-loaded PDEs was visualized by live/dead staining conducted by Annexin V-FITC/PI Apoptosis Detection Kit and showed that the ^A^GE@Cur treatment exhibited superior tumor cell killing effect (Figure 3D).

### 3.3 Inhibition of macrophages migration

**FIGURE 4:**
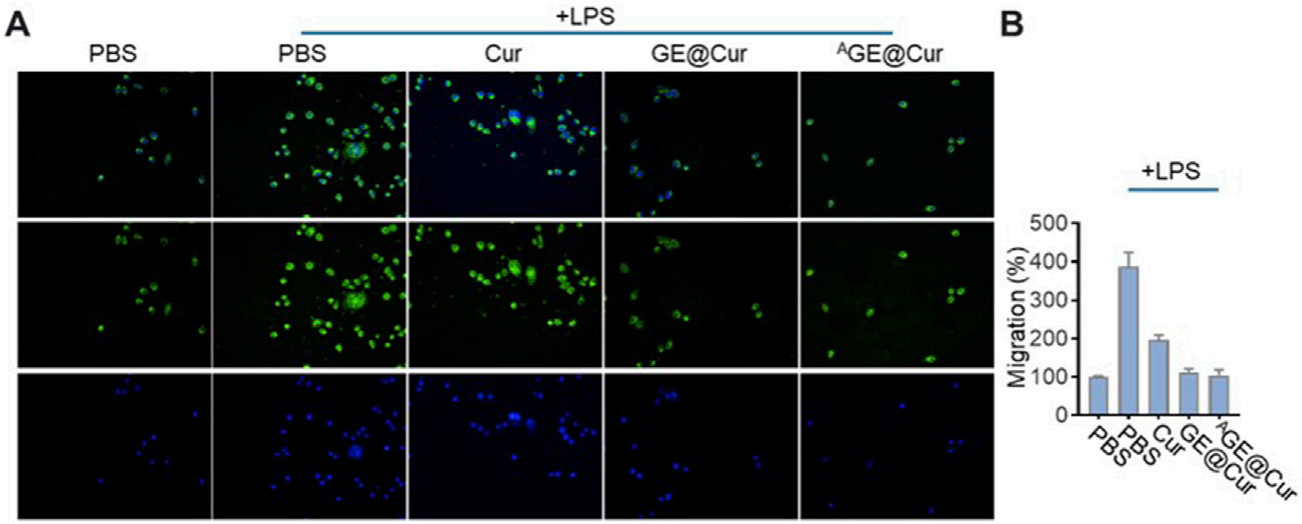
**(A)** Transwell-Matrigel invasion assay of Raw 264.7 cells treated with different formulations. **(B)** Migration analysis of cells treated with different formulations in the Transwell-Matrigel invasion assay.

The aggregation of macrophages has the potential to amplify inflammatory responses and promote tumor proliferation and metastasis (Qian and Pollard, 2010). Consequently, mitigating the recruitment of macrophages represents a promising therapeutic approach for the management of inflammation-mediated disorders and oncological conditions. Cur has been reported as holding the potential in inhibiting the migration of the macrophages (Zhong et al., 2022). As shown in Figure 4, LPS-activated RAW264.7 macrophages tend to move from the upper chamber to the lower chamber in the Transwell assay. The addition of GE@Cur and ^A^GE@Cur could largely reduce macrophage migration, demonstrating that the Cur-loaded PDEs could inhibit activated macrophage-induced migration.

### 3.4 *In vivo* anticancer therapy and microenvironment regulation

**FIGURE 5:**
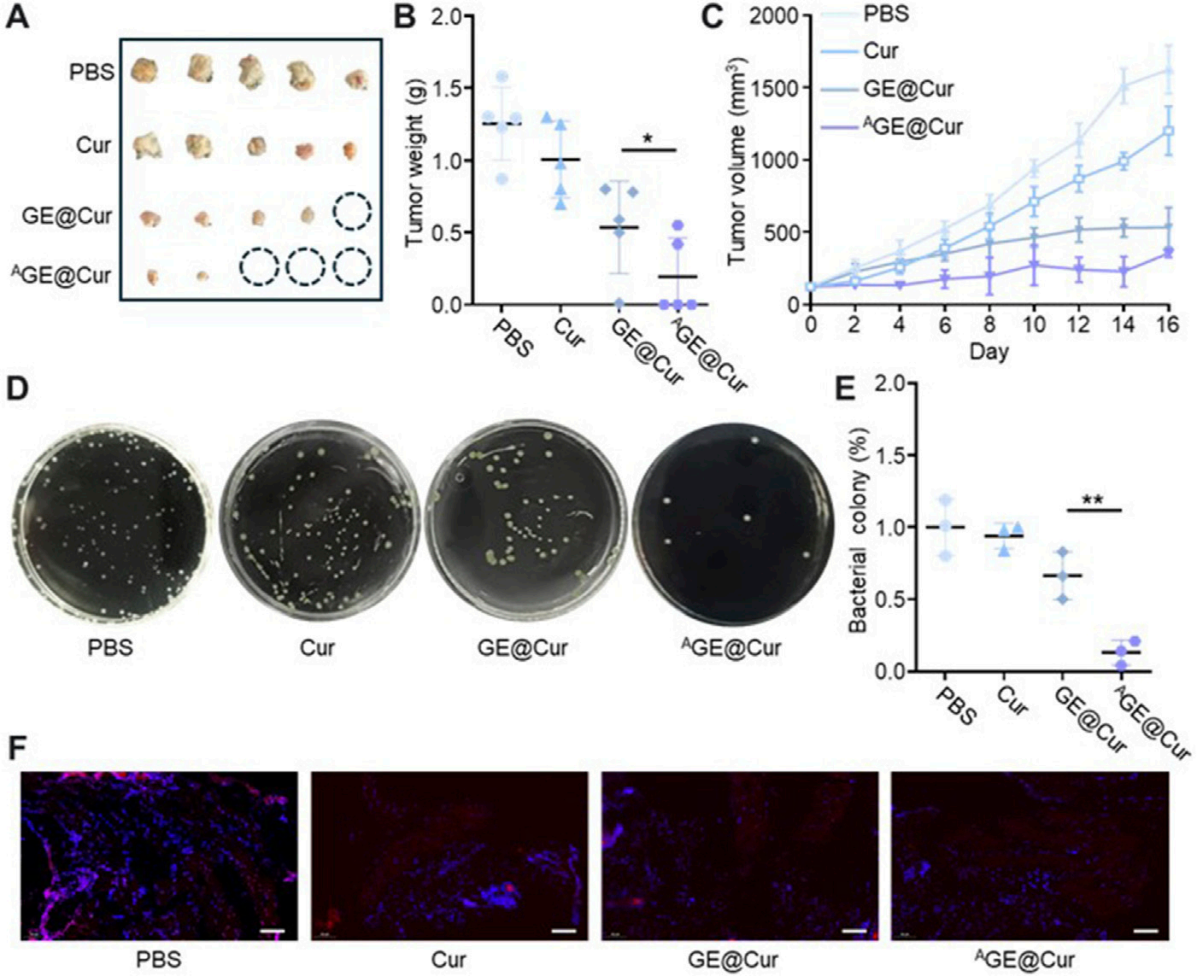
**(A)** Images of excised tumors of the different groups. **(B)** Tumor weight of excised tumors of the different groups. **(C)** Tumor volume curve of each treatment group. **(D)** Photographs of the *Escherichia coil* colonies from different treatment groups. **(E)** Counting of *Escherichia coil* colonies in tumor. **(F)** Images of immunofluorescence staining of the ROS. Nucleus was stained with DAPI. Scale bar: 50 μm. (*p < 0.05, **p < 0.01).

Encouraged by the excellent *in vitro* therapeutic performances of the ^A^GE@Cur, we next proceeded to study the *in vivo* anticancer efficacy. The synergistic effects encompassing antibacterial efficacy, tumor cell inhibition, and immunomodulatory properties of the ^A^GE@Cur are anticipated to confer an enhanced tumor suppressive outcome via targeted therapy. The *in vivo* tumor suppression efficiency was assessed in an *E. coil*-infected ID8 tumor model. The *E. coil* infected tumor on mouse model was treated with PBS, Cur, GE@Cur, and ^A^GE@Cur (based on 10 mg Ber/kg body weight) by tail vein injection, respectively. The administration was conducted every other day for 3 treatments on Day 0, Day 2, and Day 4. Tumors in the PBS control demonstrated a precipitous growth trajectory over a period of 16 days. In contrast, the ^A^GE@Cur treatment significantly impeded tumor proliferation, aligning with the *in vitro* cytotoxicity assessments. As illustrated in Figures 5A, B, the mean tumor weight following ^A^GE@Cur was substantially reduced when compared to the PBS control group. Comparable outcomes were observed in tumor volume measurements (Figure 5C). The number of bacteria in the tumor has also been effectively controlled under the treatment of the ^A^GE@Cur (Figures 5D, E). The robust ROS levels response as a marker and immunological defense mechanism in tumor. Next, we conducted an immunofluorescence analysis of the ROS levels within the tumor tissue post-various treatments. As shown in Figure 5F, a marked reduction in ROS levels was evident following ^A^GE@Cur treatment, indicative of the potent anti-infective and immunomodulatory properties afforded by the targeted PDEs. In addition, the body weight of mice during the treatment were similar and steadily increased, providing strong evidence for the biosafety of the drug-loaded PDEs (Supplementary Figure S4). The above results suggested that the ^A^GE@Cur hold exceptional therapeutic effects in caner treatment.

## 4 Conclusion

In conclusion, we successfully address the challenges associated with the application of EXOs and the treatment of ovarian cancer by developing a novel therapeutic strategy utilizing ginger-derived PDEs. The constructed multifunctional microenvironment nanoregulator, namely ^A^GE@Cur, demonstrates effective tumor inhibition effect through the remodeling of the tumor microenvironment. By leveraging the advantages of ginger-derived PDEs, including high encapsulation efficiency of Cur and tumor-targeted modification with aptamers, this study overcomes the biosafety concerns and application limitations of animal-derived exosomes. The multifaceted therapeutic platform presented here not only provides a promising approach for targeted ovarian cancer treatment but also paves the way for the development of innovative microenvironment nanoregulators that could be translated into clinical applications. This research underscores the potential of plant-derived exosomes as a safe and effective modality in the fight against ovarian cancer and other malignancies.

## Data Availability

The original contributions presented in the study are included in the article/Supplementary Material, further inquiries can be directed to the corresponding authors.
