# Supplementary material for: A multifaceted microenvironment nanoregulator for targeted ovarian cancer therapy
Source: Front Pharmacol. 2025 Mar 27;16:1584463. doi: 10.3389/fphar.2025.1584463 (PMC12000774; doi:10.3389/fphar.2025.1584463)
Supplement: Supplementary file 1 [file DataSheet1.docx]

Supplementary Material

**A multifaceted microenvironment nanoregulator for targeted ovarian cancer therapy**

Yizheng Zu^1,2+^, Min Li^1,2+^, Ruyue Li^1,2^, Shaohan Ma^1,2^, Yu’e Yang^1,2^, Shun Zhang^3^, Yuan Ma^*1,2^, Tiantian Wu^*3^, Chunfang Ha^*1,2^

^1^General Hospital of Ningxia Medical University, Yinchuan, 750004, China

^2^Key Laboratory of Fertility Preservation & Maintenance of Ministry of Education, Ningxia Medical University, Yinchuan, 750004, China

^3^School of Pharmacy, Hainan Medical University, Haikou, China.

^+^These authors contributed equally to this work.

*** Correspondence:**Yuan Ma

546840463@qq.com

Tiantian Wu

hy0207149@muhn.edu.cn

Chunfang Ha

hachunfang@163.com

# Supplementary Figures


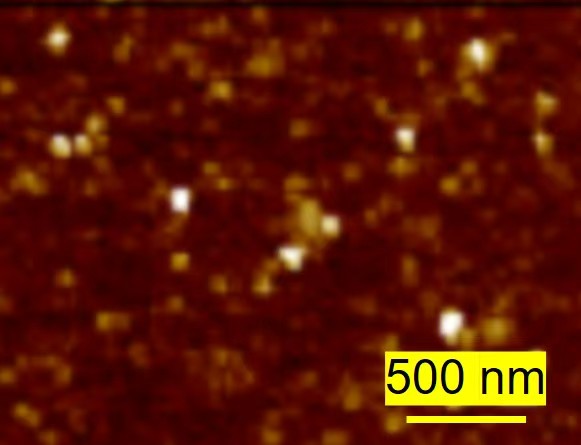


**Supplementary Figure 1.** AFM images of the GE. Scale bar: 500 nm.


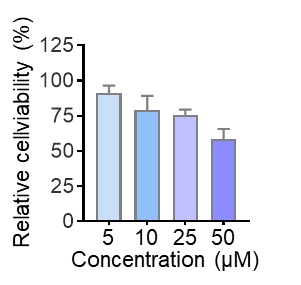


**Supplementary Figure 2.** Relative cellviability of ID8 cell after the treatment of different concentration of Cur.


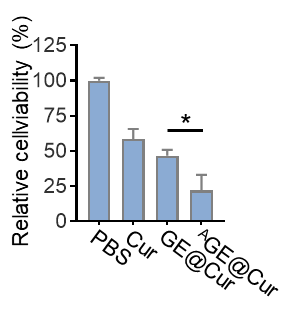


**Supplementary Figure 3.** Cell viability analysis of cells after the treatment with different formulations.


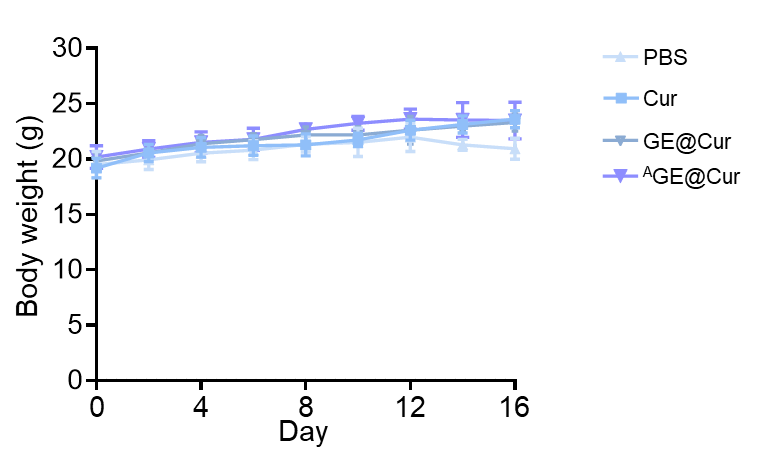


**Supplementary Figure 4.** Body weight curve of each treatment group.
